# Methylation of N6 adenosine‐related long noncoding RNA: effects on prognosis and treatment in ‘driver‐gene‐negative’ lung adenocarcinoma

**DOI:** 10.1002/1878-0261.13323

**Published:** 2022-11-16

**Authors:** Hao‐Shuai Yang, He‐Yuan Cai, Shi‐Chao Shan, Ting‐Fei Chen, Jian‐Yong Zou, Maimaiti Abudurufu, Hong‐He Luo, Yi‐Yan Lei, Zun‐Fu Ke, Ying Zhu

**Affiliations:** ^1^ Department of Thoracic Surgery The First Affiliated Hospital, Sun Yat‐sen University Guangzhou China; ^2^ Department of Pathology & Institution of Precision Medicine The First Affiliated Hospital, Sun Yat‐sen University Guangzhou China; ^3^ Department of Radiology The First Affiliated Hospital, Sun Yat‐sen University Guangzhou China

**Keywords:** driver‐gene‐negative, long noncoding RNA, lung adenocarcinoma, N6 adenosine methylation, nomogram

## Abstract

The improvement of treatment for patients with ‘driver‐gene‐negative’ lung adenocarcinoma (LUAD) remains a critical problem to be solved. We aimed to explore the role of methylation of N6 adenosine (m6A)‐related long noncoding RNA (lncRNA) in stratifying ‘driver‐gene‐negative’ LUAD risk. Patients negative for mutations in *EGFR*, *KRAS*, *BRAF*, *HER2*, *MET*, *ALK*, *RET*, and *ROS1* were identified as ‘driver‐gene‐negative’ cases. RNA sequencing was performed in 46 paired tumors and adjacent normal tissues from patients with ‘driver‐gene‐negative’ LUAD. Twenty‐three m6A regulators and relevant lncRNAs were identified using Pearson's correlation analysis. K‐means cluster analysis was used to stratify patients, and a prognostic nomogram was developed. The CIBERSORT and pRRophetic algorithms were employed to quantify the immune microenvironment and chemosensitivity. We identified two clusters highly consistent with the prognosis based on their unique expression profiles for 46 m6AlncRNAs. A risk model constructed from nine m6A lncRNAs could stratify patients into high‐ and low‐risk groups with promising predictive power (C‐index = 0.824), and the risk score was an independent prognostic factor. The clusters and risk models were closely related to immune characteristics and chemosensitivity. Additional pan‐cancer analysis using the nine m6AlncRNAs showed that the expression of DIO3 opposite strand upstream RNA (*DIO3OS*) is closely related to the immune/stromal score and tumor stemness in a variety of cancers. Our results show that m6AlncRNAs are a reliable prognostic tool and can aid treatment decision‐making in ‘driver‐gene‐negative’ LUAD. DIO3OS is associated with the development of various cancers and has potential clinical applications.

## Graphical abstract

Based on RNA sequencing results for patients with ‘driver‐gene‐negative’ lung adenocarcinoma (LUAD), m6A‐related lncRNAs were found and experimentally verified to have good predictive efficacy for the prognosis and immune characteristics of patients with ‘driver‐gene‐negative’ LUAD. DIO3OS as a key lncRNA was found to affect tumor cell stemness in a variety of cancers.

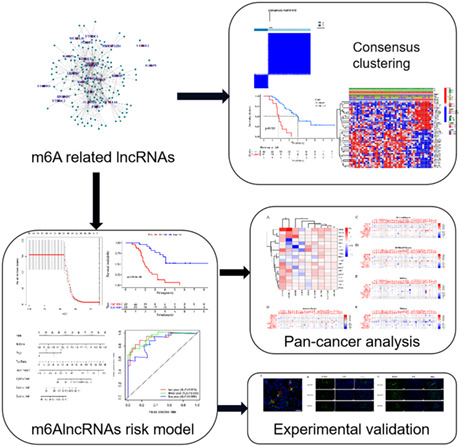

**Abbreviations**
ALK: Anaplastic Lymphoma kinase
AUC: under the curve
DIO3OS: DIO3 opposite strand upstream RNA
EGFR: epidermal growth factor receptor
FFPE: formalin‐fixed paraffin‐embedding
FISH: fluorescence *in situ* hybridization
Her2: human epithelial growth factor receptor‐2
IC‐50: half‐maximal inhibitory concentration
lncRNA: long noncoding RNA
LUAD: lung adenocarcinoma
m6A: methylation of N6 adenosine
MET: human epithelial growth factor receptor‐2
OS: overall survival
ROC: receiver operating characteristic
ROS1: ROS proto‐oncogene 1
TCGA: The Cancer Genome Atlas

## 1 Introduction

Lung cancer is one of the most prevalent cancers and the leading cause of cancer‐related deaths [1]. Lung adenocarcinoma (LUAD) is the most frequent histological manifestation of lung cancer, accounting for > 40% of all cases [2]. With the continuous development of genetic diagnosis technology, a series of driver genes that play an important role in the occurrence and development of lung cancer [3, 4], including EGFR, ALK, KRAS, MET, BRAF, ROS1, RET, and HER2 [5, 6, 7], have been discovered. The application of driver gene‐targeted drugs has improved the prognosis of patients with positive driver gene mutations [8, 9], while the improvement of treatment for driver‐gene‐negative patients remains a critical problem to be solved [10, 11].

The methylation of N6 adenosine (m6A) is the most common internal post‐transcriptional modification of RNAs in many diseases [12]. Previous research has revealed that m6A methylation is a dynamically reversible biological process regulated by a combination of methyltransferases (writers), related reading proteins (readers), and demethylases (erasers) [13]. Previous studies have shown that m6A and its related long noncoding RNAs (lncRNAs) play an important role in the development of lung cancer [14, 15] and are expected to be new therapeutic sites [16]. However, its role in ‘driver‐gene‐negative’ LUAD remains unknown.

In this study, we aimed to explore the role of m6A‐related lncRNAs in patients with ‘driver‐gene‐negative’ LUAD, establish a trustworthy prognosis prediction model based on m6A‐related lncRNAs, and conduct in‐depth studies at the pan‐cancer level. Pan‐cancer analysis of key lncRNAs was also carried out in 33 cancer types from The Cancer Genome Atlas (TCGA) database to explore the possibility of acting as a marker in multiple tumors.

## 2 Materials and methods

### 2.1 Determination of patients with ‘driver‐gene‐negative’ LUAD and acquisition of clinical data

A total of 784 patients with EGFR mutation‐negative LUAD who were not targeted for current mainstream targeted therapy between September 2003 and June 2015 at the First Affiliated Hospital of Sun Yat‐sen University (FAHSYSU) were included in this study. The study methodology conformed to the Declaration of Helsinki criteria, and the experiments were performed with the understanding and written consent of the subjects. This project was approved by the Ethics Committee and Institutional Review Board of Sun Yat‐sen University as No. 2021‐531, and the requirement for informed consent was waived. The mutation status of the patient's tumor driver genes was determined by the following process. First, we examined tissue samples from 784 patients with pathologically confirmed EGFR mutation‐negative LUAD using a 13‐genes panel (including EGFR, KRAS, BRAF, PIK3CA, NRAS, HER2, MET, AKT1, c‐KIT, PDGFRA, ALK, RET, and ROS1) to determine the expression of their driver genes. EGFR and KRAS were validated in patients using ARMSPCR, and ALK was validated using FISH. Finally, we identified 371 patients with LUAD as ‘driver‐gene‐negative’ with EGFR, ALK, KRAS, MET, BRAF, ROS1, RET, and HER2 mutation negative, which could not utilize current mainstream targeted therapy [5]. The patients included in this study were part of the previous study cohort of our team [5]. Clinical information, including sex, age, tumor TNM stage, and differentiation, was collected. Tumor staging was based on the American Joint Committee on Cancer 7th Edition Cancer Staging System. OS was measured from the date of surgery to the date of death or the last date of follow‐up. For all patients, our follow‐up lasted for at least 5 years unless the patient died.

### 2.2 Samples selection and genetic data acquisition

A total of 60 paired tumor and precancerous tissue samples were selected randomly from 371 patients with ‘driver‐gene‐negative’ LUAD in FAHSYSU, which includes 15 pairs with stage I, 15 pairs with stage II, 15 pairs with stage III, and 15 pairs with stage IV. However, 14 pairs were excluded, of which eight pairs were excluded due to degradation of RNA during storage and six pairs due to missing partial clinical information. The clinical information of the 46 patients is presented in Table 1 and Table S1. Total RNA was harvested from fresh tissue, RNA was analyzed using a Nanodrop2000 spectrophotometer (Thermo Fisher Scientific, Waltham, MA, USA), and RNA integrity was assessed with an Agilent 2100 Bioanalyzer system (Agilent Technologies, Santa Clara, CA, USA). Sample labeling and array hybridization were performed according to the Agilent One‐Color Microarray Gene Expression Analysis Protocol (Agilent Technologies). Next, 100 μL of hybridization solution was dispensed into spacer slides and assembled into gene expression microarray slides. We used the 4× 44 K whole human genome expression microarray (Agilent design ID 026652, GEO accession number GPL13497) to obtain expression profiles of 27 958 genes in the remaining 46 pairs. We used Gene Spring GX v12.1 software package to perform quantile normalization and subsequent data processing, and genes with low expression or close to the background level were excluded from the analyses.

**Table 1:** Clinical information of included 46 patients with ‘driver‐gene‐negative’ LUAD.

Variables	Number of patients (%)
Status	
Dead	13 (28.26)
Alive	33 (71.74)
Gender	
Male	22 (47.83)
Female	24 (52.17)
Age	
< 60	28 (60.87)
≥ 60	18 (39.13)
Clinical stage	
I	13 (28.26)
II	13 (28.26)
III	11 (23.91)
IV	9 (15.57)
T stage	
T1	21 (45.65)
T2	17 (36.96)
T3	4 (8.70)
T4	4 (8.70)
N stage	
N0	19 (41.30)
N1	9 (15.57)
N2	12 (26.09)
N3	6 (13.04)
M stage	
M0	38 (82.61)
M1	8 (17.39)
Overall survival	
Years (mean ± SD)	3.13 ± 1.62

### 2.3 Identification of m6A genes and related lncRNAs and cluster analysis

Based on previous studies, we identified 23 m6A genes in the genetic information of 46 patients with ‘driver‐gene‐negative’ LUAD and extracted their expression matrix, which contained eight ‘writers’ (METTL3, METTL14, METTL16, WTAP, VIRMA, RBM15, RBM15B, and ZC3H13), 13 ‘readers’ (YTHDC1, YTHDC2, YTHDF1, YTHDF2, YTHDF3, IGFBP1, IGFBP2, IGFBP3, HNRNPC, FMR1, LRPPR, HNRNPA2B1, and RBMX), and two ‘erasers’ (FTO, ALKBH5) [13]. We used Pearson's correlation analysis to identify relevant lncRNAs, with the criteria [17] of | Pearson |*R*| > 0.4 and *P* < 0.001. The lncRNAs were selected when they correlated with the expression of at least one of the genes; finally 154 m6AlncRNAs were identified.

K‐means algorithm was used to stratify patients as different clusters, and the process performed the classification number *K* = 2–9. The r package ‘ConsensuClusterPlus’ was used to perform the steps above 1000 times to guarantee the stability of classification.

### 2.4 Establishment and validation of risk assessment model based on m6AlncRNAs

We performed univariate cox regression on 154 previously identified m6AlncRNAs, with the criteria of *P* < 0.05. The 46 key m6AlncRNAs, which were identified as prognosis‐related, was used to construct the model by LASSO regression. Eventually, nine m6AlncRNAs were included in the model, that is, FMO6P, SDHAP1, C9orf163, MBL1P, DIO3OS, CSNK1A1P1, UBE2Q2P1, BCORP1, and ZDHHC8P1. The formula used to calculate the risk score using the constructed model was as follows:

$$\text{Risk Score}={\hat{h}}_{0}\left(t\right)\sum_{i=1}^{k}\beta \mathrm{iSi}.$$

The median risk score was used to stratify the patients into high‐risk and low‐risk groups. The score distribution dot plot, survival status dot plot, and gene expression heatmap were plotted to validate this risk model. The process was visualized using the r packages ‘survival’, ‘glmnet’, ‘pbapply’, ‘survivalROC’, ‘survminer’, and ‘pHeatmap’.

### 2.5 Exploration in the immunotherapeutic and chemotherapeutic treatment

Based on the CIBESORT deconvolution algorithm, we explored the infiltration of immune cells and key checkpoint genes (PD‐1 and PD‐L1) expression at different groups (normal and tumor, Cluster 1 and Cluster 2, and high‐ and low‐risk group). The half‐maximal inhibitory concentration (IC50) of chemotherapeutic drugs was predicted by using the ‘pRRophetic’ package in r [18]. This package predicted IC50 by establishing statistical models based on drug sensitivity and gene expression data from the Genomics of Drug Sensitivity in Cancer website (www.cancerrxgene.org/). This was visualized using the r package ‘ggplot2’.

Immunohistochemistry was used to analyze PD‐1 and PD‐L1 expression between high‐ and low‐risk group patients. After routine sectioning, the slides were deparaffinized and dehydrated with graded alcohol, blocked with endogenous peroxidase for inactivation, antigen retrieval, and blocked with goat serum. Primary antibody was added at 4 °C, labeled secondary antibody was added at 37 °C, then stain was added, mounted, and observed with a microscope. Proportion of positive cells is graded as four grades based on staining ratio of < 10%, 10–49%, 50–74%, 75–100%, which were scored to compare the differences between the two groups.

### 2.6 Pan‐cancer analysis of key m6AlncRNA

To further investigate the roles of the key m6AlncRNAs in other cancers, we performed a pan‐cancer analysis of 33 cancer species contained in TCGA. The RNA‐seq (HTSeq–FPKM) and mutation data of 33 cancers were downloaded from the University of California, Santa Cruz Xena database (https://xena.ucsc.edu/, originating from the TCGA database). The expression of key m6AlncRNAs is shown as a heatmap with logFC designed using the r package ‘pheatmap’. Using the ESTIMATE algorithm, we calculated stromal and immune cell scores in pan‐cancer tissues to forecast tumor purity and infiltrating conditions of stromal/immune cells. The RNA‐based stemness scores (RNAss) and DNA methylation‐based stemness scores of patients were calculated using RNA expression and DNA methylation [19]. The process is implemented by r packages ‘estimate’, ‘cor. Test’, ‘limma’, and ‘corrplot’.

### 2.7 Fluorescence *in situ* hybridization (FISH) of DIO3OS

Formalin‐fixed paraffin‐embedding (FFPE) specimens from five patients with ‘driver gene‐negative lung adenocarcinoma’ were randomly selected from high‐ and low‐risk groups, and 5 FAM‐labeled DIO3OS FISH and FISH Kit (Axl‐bio, Guangzhou, China) were used following the manufacturer's instructions. Digital Sight DS‐FI2 confocal microscope capture (Nikon, Japan) was used to the images.

### 2.8 Statistical analysis

Univariate and multivariate Cox regression analyses were used to evaluate the correlations between various factors, including independent prognostic analysis and variable selection included in the nomogram and model. One‐way analysis of variance and Kruskal–Wallis tests were used to compare the differences between three or more groups. Kaplan–Meier survival analysis was used to explore survival differences between the groups. The Wilcoxon signed‐rank test was used to compare the difference in immune cell infiltration between the groups, and Spearman correlation analysis was used to analyze the correlation between immune cell infiltration and risk score. Predicting efficacy of models presented as the area under the receiver operating characteristic curves (AUCs). Time‐dependent receiver operating characteristic (ROC) curves of the risk model and nomogram were plotted. All data were processed using r version 4.0.5.

## 3 Results

### 3.1 Identification of m6AlncRNAs in 46 patients with ‘driver‐gene‐negative’ LUAD

**Fig. 1:**
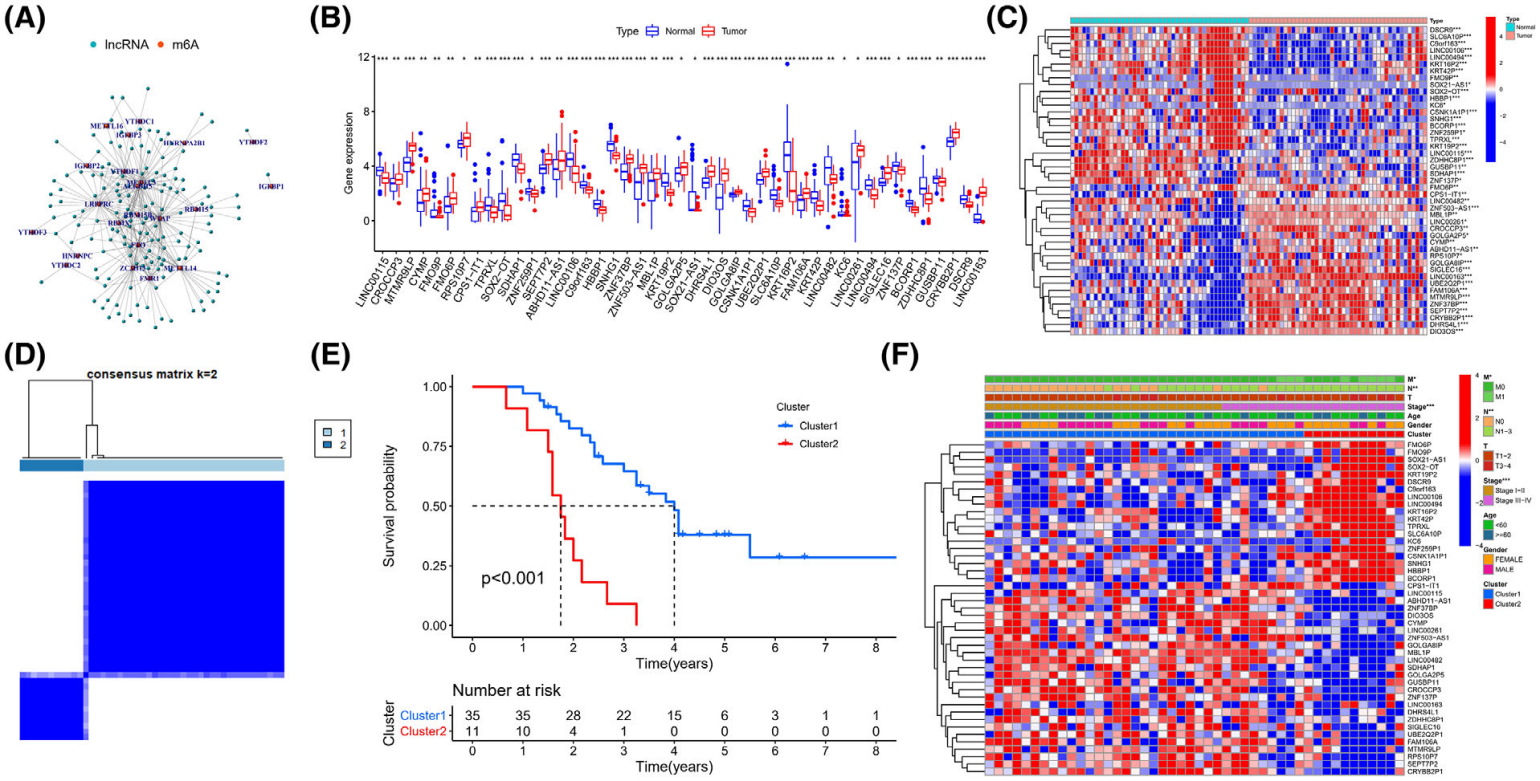
Prognosis‐related m6AlncRNAs in 46 patients with ‘driver gene negative’ LUAD. (A) Coexpression network of lncRNAs and m6A‐related genes based on Pearson correlation analysis. (B, C) 46 prognosis‐related m6AlncRNAs expression in normal and tumor tissue compared by Wilcoxon Rank test with a threshold of *P* < 0.05. Data are presented as quartiles, and error bars indicate extreme values. (D) Patients were classified into Cluster 1 (*n* = 35) and Cluster 2 (*n* = 11) by consensus clustering. (E) Kaplan–Meier curves of OS in Cluster 1 and Cluster 2, the log‐rank test was used to assess the difference between groups. (F) Heatmap and clinical characteristics in Cluster 1/2. LUAD lung adenocarcinoma; OS overall survival. *P < 0.05, **P < 0.01, and ***P < 0.001.

We obtained RNA‐seq data from 46 ‘driver gene‐negative’ LUAD patients, consisting of 537 lncRNAs and 17 060 mRNAs. Based on Pearson correlation analysis of coexpression, 154 m6AlncRNAs (m6AlncRNAs) related to 23 m6A‐related genes were identified, and a coexpression network of m6AlncRNAs was constructed (Fig. 1A). Finally, 46 different expressed m6AlncRNAs were identified as prognosis‐related by Wilcoxon Rank test and univariate Cox regression analysis (*P* < 0.05) (Fig. 1B,C).

### 3.2 Clustering classifications of 46 m6AlncRNAs and clinical correlation analysis

K‐mean cluster analysis was performed on the expression levels of 46 m6AlncRNAs according to the classification number *K* = 2–9, and the results showed that *K* = 2 was the best classification, including Cluster 1 (*n* = 35) and Cluster 2 (*n* = 11) (Fig. 1D). Next, a Kaplan–Meier survival analysis of prognosis was explored between Cluster 1 and Cluster 2 patients, and it was found that the OS of Cluster 1 were better than those of Cluster 2 (*P* < 0.001) (Fig. 1E). In addition, clinical correlation analysis showed that tumor stage, N stage, and M stage were correlated with clusters. Compared to Cluster 2, patients in Cluster 1 had an earlier tumor stage, no lymph node metastasis, or distant metastasis, and there was no significant difference in age and sex between the two clusters (Fig. 1F).

### 3.3 Signature constructed from nine m6AlncRNAs can predict prognosis of patients with ‘driver‐gene‐negative’ LUAD

**Fig. 2:**
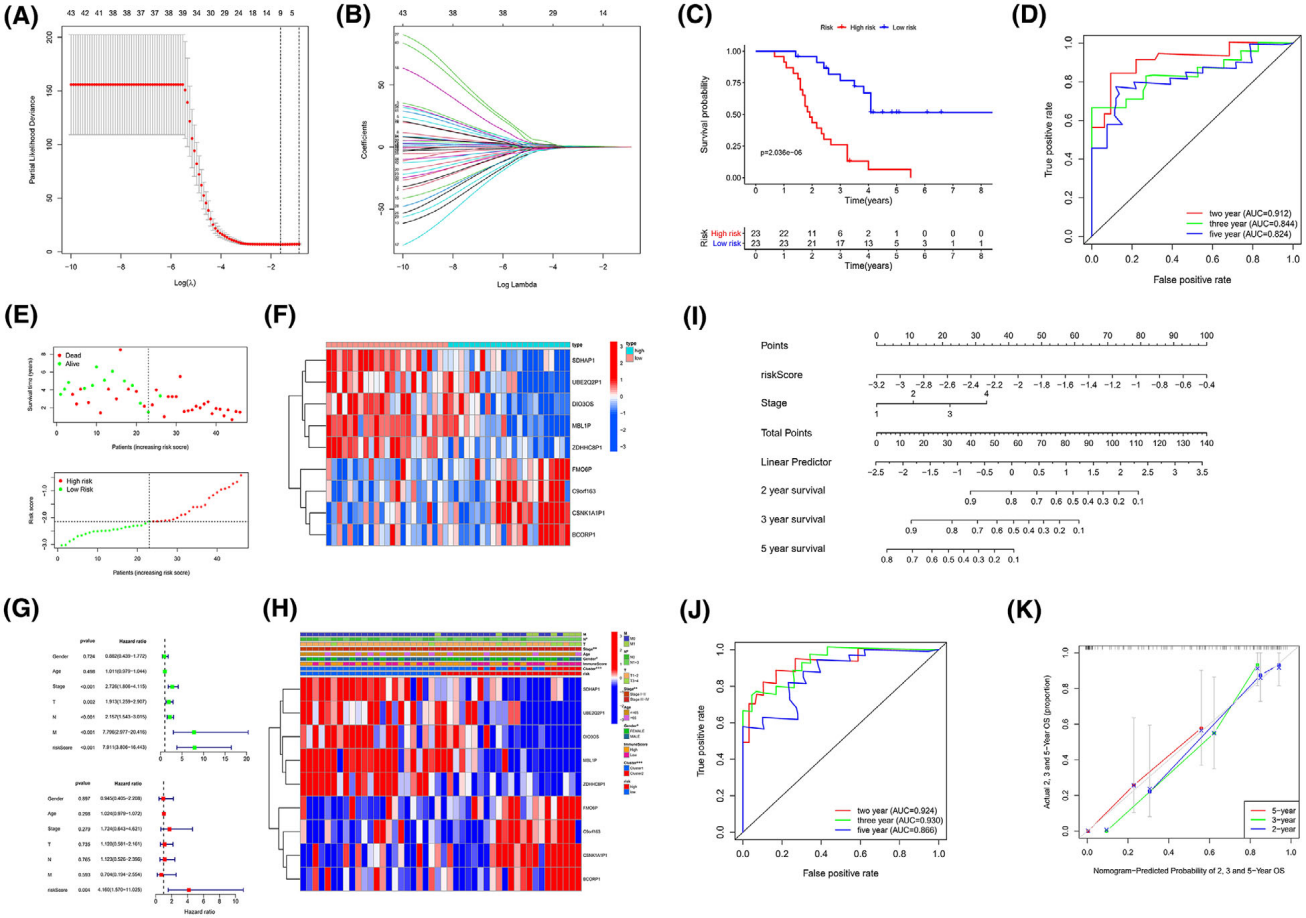
Construction of prognostic model with 9‐m6AlncRNAs signature of 46 patients with ‘driver‐gene‐negative’ LUAD. (A, B) LASSO regression and the selection of minimum criteria. (C) Kaplan–Meier curves of OS in low‐risk (*n* = 23) and high‐risk (*n* = 23) group, the log‐rank test was used to assess the difference between groups. (D) Time‐dependent ROC curves of prognostic model. The model is highly powered but lacks appropriate external validation. (E, F) Distribution of risk score and survival status and heatmap of the nine prognostic m6AlncRNAs. (G, H) Forest plot of univariate/multivariate Cox proportional risk regression and heatmap of clinical characteristics in low‐risk and high‐risk groups, the error bars indicate the 95% confidence interval. (I) Nomogram based on risk score and tumor stage. (J) Time‐dependent ROC curves of 2, 3, and 5 years in Nomogram. (K) The efficacy of nomogram was estimated by calibration curves, and the error bars indicate the 95% confidence interval. LASSO, Least absolute shrinkage and selection operator; LUAD, lung adenocarcinoma; OS, overall survival; ROC, receiver operating characteristic. *P < 0.05, **P < 0.01, and ***P < 0.001.

To construct the m6AlncRNAs signature to predict the prognosis, we performed LASSO regression analysis on the m6AlncRNAs obtained above to determine the best genes for building the model (Fig. 2A,B). Finally, nine m6AlncRNAs were identified and a model was constructed using multivariate Cox proportional risk regression analysis. The formula of m6AlncRNAs signature was as follows:

$$\text{risk score}=\begin{array}{l} 0.099824^{*}\;\mathrm{FMO}6P-0.27839^{*}\;\text{SDHAP1}\\ +\;0.004455^{*}C9\mathrm{orf}163\\ -\;0.03363^{*}\;\mathrm{MBL}1P-0.08103^{*}\;\mathrm{DIO}3\mathrm{OS}\\ +\;0.007434^{*}\;\text{CSNK}1A1\text{P1}\\ -\;0.22867^{*}\;\mathrm{UBE}2Q2\text{P1}\\ +\;0.283377^{*}\;\text{BCORP1}\\ -\;0.17081^{*}\;\text{ZDHHC}8\text{P1. } \end{array}$$

The low‐risk group (*n* = 23) and high‐risk group (*n* = 23) were selected according to the median risk score, which was calculated using the formula in 46 patients with LUAD. The Kaplan–Meier survival curve of the two groups showed that the low‐risk group had a better survival outcome at 3‐ and 5‐year OS than the high‐risk group (Fig. 2C), while the distribution plot and survival status plot of the risk score also showed that the low‐risk group had a better survival outcome (Fig. 2E,F). The time‐dependent ROC curves suggested that the AUCs of the 2‐, 3‐, and 5‐year signatures were 0.912, 0.844, and 0.824, respectively (Fig. 2D). All these results proved the m6AlncRNAs signature can effectively predict the prognosis of 46 patients with ‘driver‐gene‐negative’ LUAD.

### 3.4 Risk score is an independent risk factor for prognosis of 46 patients with ‘driver‐gene‐negative’ LUAD

First, we performed a Cox proportional risk regression analysis to prove that the risk score was an independent risk factor for prognosis. Univariate Cox regression analysis indicated that tumor stage, T stage, N stage, M stage, and risk score were associated with the prognosis of LUAD patients, while risk score was the only independent risk factor for prognosis by multivariate Cox regression analysis (Fig. 2G). Second, we analyzed correlations between the two groups; and clinical characteristics, tumor stage, N stage, sex, and clusters were correlated with different groups. Compared with the high‐risk group, the low‐risk group had an earlier tumor stage and less lymph node metastasis, which were all assigned to Cluster 1. More information regarding these correlations is shown in Fig. 2H. Finally, correlations between risk score and clinical characteristics were further performed in patients with LUAD, which showed that Cluster 1, early stage (Stage I–II), T1‐2, N0, and M0 had lower risk scores, while Cluster 2, advanced stage (Stage III–IV), T3‐4, N1‐3, and M1 had higher risk scores. We found that the risk score was independent of the number of circulating tumor cells in the sample, which may further explain the better prognosis in the low‐risk group.

### 3.5 Construction and validation of nomogram for predicting prognosis of 46 patients with ‘driver‐gene‐negative’ LUAD

We constructed a nomogram to improve the predictive power of the m6AlncRNAs signature and facilitate the clinical application of the model. Although the risk score was the only independent prognostic factor, we included it in the nomogram because of the important role of stage and high predictive power in clinical work. Finally, a nomogram combining the risk score with the tumor stage was constructed (Fig. 2I). The 2‐, 3‐, and 5‐year AUC of ROC curves of nomogram significantly improved to 0.924, 0.930, and 0.866, respectively (Fig. 2J). The C‐index of the nomogram was 0.829, and the calibration curve showed that the three calibration curves for OS were all close to the standard curve, which proved that the prognostic nomogram had better predictive performance (Fig. 2K).

### 3.6 Differences in immune characteristics between clusters and risk groups

**Fig. 3:**
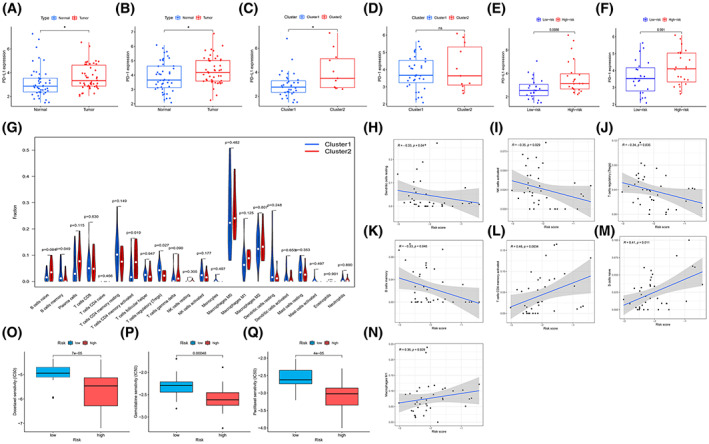
Relationship between m6AlncRNA profile and tumor immunotherapy and chemosensitivity (A–F) PD‐1/PD‐L1 different expression in different groups between normal (*n* = 46) and tumor (*n* = 46), Cluster 1 (*n* = 35) and Cluster 2 (*n* = 11), high‐ (*n* = 23) and low‐risk (*n* = 23) groups by Wilcoxon Rank test with a threshold of *P* < 0.05. (G) The infiltration of immune cells in Cluster 1 and Cluster 2 by CIBESORT with Wilcoxon signed‐rank test. (H–N) Correlations between risk score and seven types of immune cells by Spearman correlation analysis. (O–Q) Drug (docetaxel, gemcitabine, and paclitaxel) sensitivity analysis between high‐ and low‐risk groups. Data are presented as quartiles, error bars indicate extreme values. *P < 0.05, **P < 0.01, and ***P < 0.001.

Immune characteristics analysis of 46 patients with ‘driver‐gene‐negative’ LUAD was performed in different clusters and risk groups. Compared to normal tissues, PD‐L1 and PD‐1 were highly expressed in tumor tissues (*P* < 0.05; Fig. 3A,B). We found that PD‐L1 expression was higher in Cluster 2 and high‐risk groups, while PD‐1 expression was not different between groups, which was consistent with the results in the two clusters (Fig. 3C–F). Immunohistochemical results also showed that the expression level of PD‐L1 and PD‐1 in high‐risk group patients was higher than in the low‐risk group; some of these sections are presented in the Fig. S1.

Furthermore, we applied the CIBRERSORT algorithm to assess the infiltration of 20 immune cells into the different subtypes of the microenvironment. Memory B cells and regulatory T cells (Tregs) were highly expressed in Cluster 1 and negatively correlated with the risk score. CD4 memory cell activation was highly expressed in Cluster 2 and positively correlated with the risk score. Other immune cells, such as NK cells, dendritic cells, naïve B cells, and M1 macrophages, were also strongly associated with the risk fraction (Fig. 3G–N). The results reflect the consistency between the cluster and risk models; and the different immune characteristics in different clusters and risk groups might lead to different therapy options and outcomes.

### 3.7 High‐ and low‐risk groups of patients with ‘driver‐gene‐negative’ LUAD have different sensitivities to chemotherapeutic agents

Based on the Cancer Genome Project drug prediction database, the ‘pRRophetic’ package was used to analyze the prediction of response to chemotherapeutic agents for patients with ‘driver‐gene‐negative’ LUAD in the high‐risk and low‐risk groups. We found that the low‐risk group had greater sensitivity to common chemotherapeutic agents such as docetaxel, gemcitabine, and paclitaxel (Fig. 3O–Q). The benefit of chemotherapy in the low‐risk group was significantly higher than that in the high‐risk group, suggesting that this risk group has good potential for clinical application.

### 3.8 Key m6AlncRNAs are associated with the tumor microenvironment in a variety of cancers

**Fig. 4:**
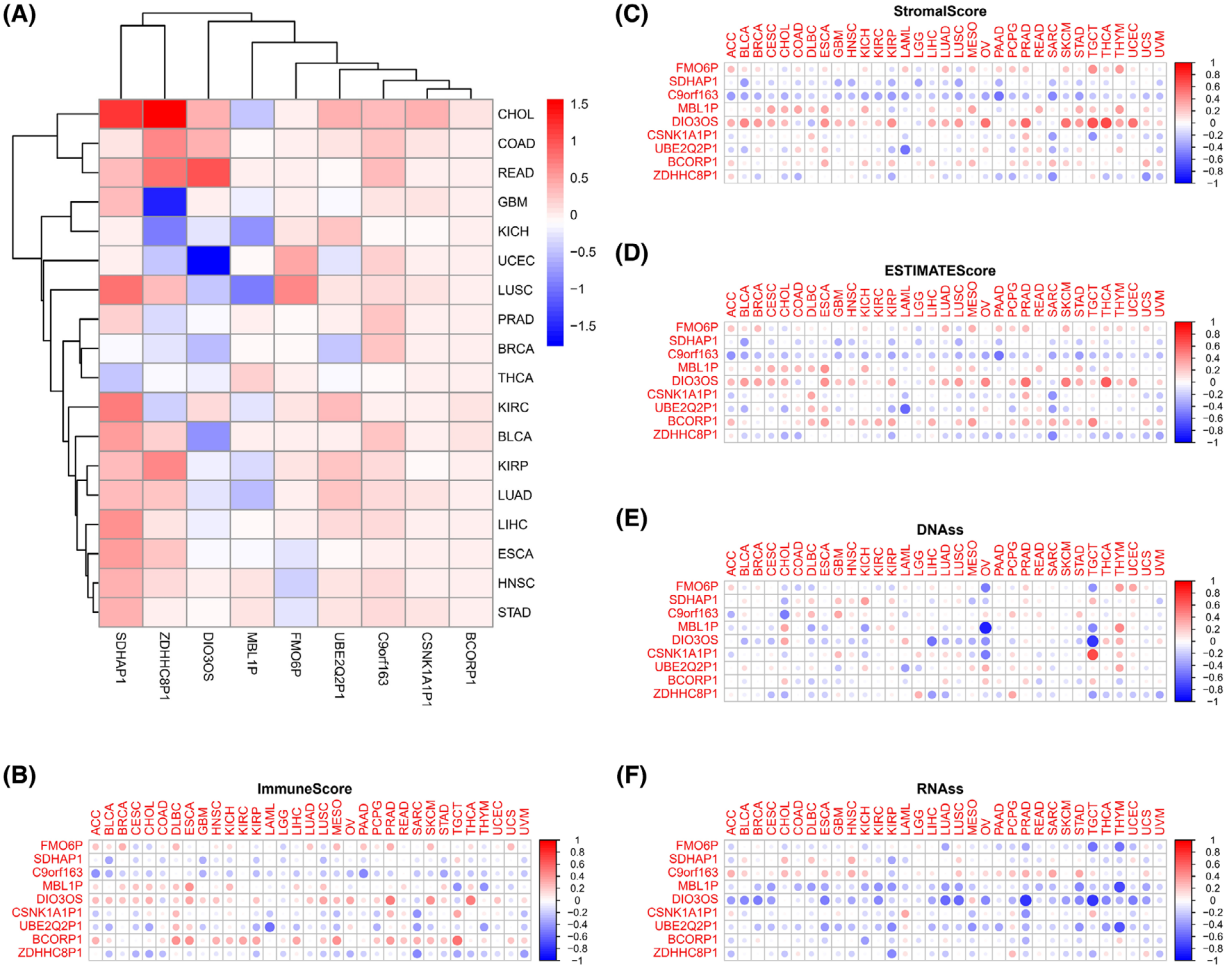
9‐m6AlncRNAs in a variety of cancers in TCGA. (A) Expression of 9‐ m6AlncRNAs in 18 cancer types with more than five normal control by Wilcoxon Rank test. (B–D) Stromal/immune score of 9‐m6AlncRNAs in 33 cancer types by Spearman correlation analysis. (E, F) Nucleic acids stemness score of 9‐ m6AlncRNAs in 33 cancer types by Spearman correlation analysis. The pan cancer analysis including ACC (*n* = 79), BLCA (*n* = 411), BRCA (*n* = 1118), CESC (*n* = 306), CHOL (*n* = 32), COAD (*n* = 471), DLBC (*n* = 48), ESCA (*n* = 162), GBM (*n* = 168), HNSC (*n* = 502), KICH (*n* = 65), KIRC (*n* = 535), KIRP (*n* = 289), LAML (*n* = 151), LGG (*n* = 529), LIHC (*n* = 345), LUAD (*n* = 527), LUSC (*n* = 501), MESO (*n* = 86), OV (*n* = 379), PAAD (*n* = 178), PCPG (*n* = 183), PRAD (*n* = 500), READ (*n* = 167), SARC (*n* = 263), SKCM (*n* = 471), STAD (*n* = 375), TGCT (*n* = 156), THCA (*n* = 512), THYM (*n* = 119), UCEC (*n* = 548), UCS (*n* = 56), UVM (*n* = 80). TCGA, The Cancer Genome Atlas.

We sought to elucidate whether the m6AlncRNAs selected in this study also play an important role in other cancer types by pan‐cancer analysis based on 33 cancer types in TCGA. The nine selected key m6AlncRNAs in some cancer species are shown in Fig. 4A, and three lncRNAs, SDHAP1, ZDHHC8P1, and DIO3OS, were significantly differentially expressed in many cancer types. We also observed a wide range in the degree of association between key m6AlncRNAs and stromal/immune scores for different cancer types. The highest correlation was observed with stromal score across cancer types. After evaluating immune/stromal scoring using ESTIMATE in different tumor types, we found that DIO3OS, C9orf163, and BL‐C were closely associated with these three scoring methods (Fig. 4B–D). At the same time, we found that DIO3OS is closely related to the tumor stemness score, especially RNAss (Fig. 4E,F).

### 3.9 Validation of expression of DIO3OS in patients with ‘driver‐gene‐negative’ LUAD

**Fig. 5:**
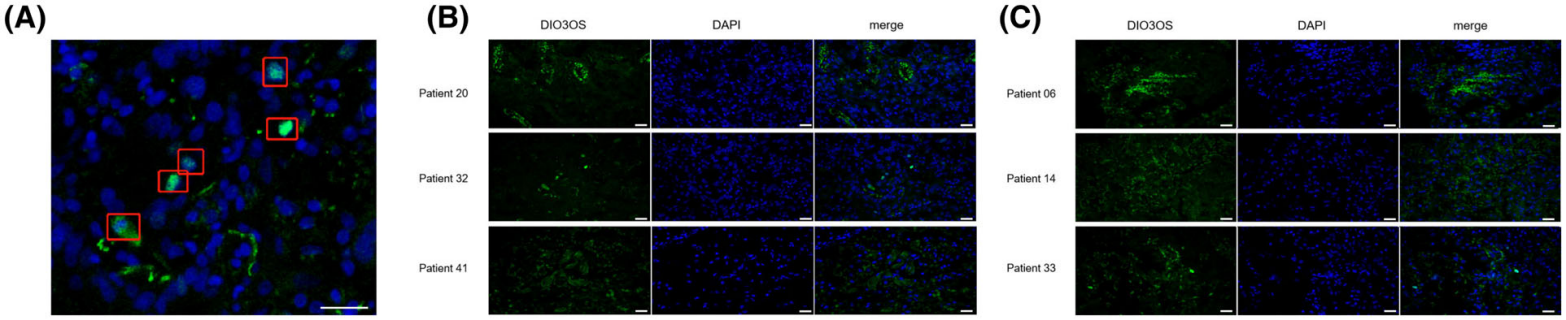
Fluorescence *in situ* hybridization (FISH) of DIO3OS in patients with ‘driver‐gene‐negative’ LUAD tissues. (A) DIO3OS is predominantly expressed in the nucleus in ‘driver‐gene‐negative’ LUAD tissues, with red boxes identifying where DIO3OS expression accumulates, Bar = 25 μm; (B) expression and distribution of DIO3OS in high‐risk patients (*n* = 3), Bar = 25 μm; (C) expression and distribution of DIO3OS in low‐risk patients (*n* = 3), Bar = 25 μm. LUAD, lung adenocarcinoma.

We validated the expression of DIO3OS in driver gene‐negative lung adenocarcinoma patients by FISH experiments, and the results showed that DIO3OS was mainly expressed in the nucleus and partially expressed in the cytoplasm (Fig. 5A). The expression of DIO3OS could be detected in all 10 randomly selected patients, and patients in the high‐risk group had lower DIO3OS expression than patients in the low‐risk group, consistent with the previous analysis results (Fig. 5B,C).

## 4 Discussion

In this study, we determined the expression of m6A‐related lncRNAs in tumor and normal samples from ‘driver‐gene‐negative’ LUAD patients. According to the expression level of m6A, a cluster and a relevant risk assessment model were constructed by relying on the key m6AlncRNAs, which can effectively distinguish the prognosis of patients and predict their tumor immune cell infiltration and chemosensitivity. In addition, we performed pan‐cancer analysis against key m6AlncRNAs and found that DIO3OS has the potential to be a therapeutic target for a variety of cancers.

Methylation of N6 adenosine is the most common RNA modification in eukaryotes, which can affect the occurrence and development of cancers by modifying mRNA and lncRNA [20, 21]. Several studies have reported that m6AlncRNAs can also be used as cancer prognostic markers [22, 23, 24]. Previous studies have shown that m6A genes regulate the development of NSCLC [25, 26, 27] and act as potential prognostic markers of NSCLC [28]. As estimated, patients with ‘driver‐gene‐negative’ LUAD account for approximately 25% of NSCLC [29, 30, 31, 32, 33]. Due to the limited treatment methods, it is often ignored in clinical work and lacks related research. Therefore, we attempted to explore the relationship between m6AlncRNAs and the prognosis of patients with ‘driver‐gene‐negative’ LUAD and new biological targets for this subtype to guide clinical treatment.

First, we identified 46 m6AlncRNAs associated with the prognosis of patients with ‘driver gene‐negative’ LUAD and further divided the patients into Cluster 1 (*n* = 33) and Cluster 2 (*n* = 11) according to k‐means clustering analysis. We found that Cluster is closely related to the clinical characteristics of patients; Cluster 1 (*n* = 33) had earlier tumor stage, N stage, M stage, and better prognosis. The two clusters showed good discrimination, suggesting that the m6AlncRNAs selected in this study may have played an important role in the development of tumors.

Second, we identified m6AlncRNAs signature that can predict the prognosis of patients with ‘driver‐gene‐negative’ LUAD, which included nine genes: FMO6P, C9orf163, CSNK1A1P1, and BCORP1, which were risk factors for prognosis, while SDHAP1, MBL1P, DIO3OS, UBE2Q2P1, and ZDHHC8P1 were protective factors. Previous studies have shown that these nine genes play important roles in tumor development. FMO6P was found to be associated with the prognosis of squamous cell lung carcinoma patients [34], SDHAP1 could promote the apoptosis of ovarian cancer [35], C9orf163 was associated with the prognosis of pancreatic cancer patients [36], DIO3OS could regulate the occurrence and development of liver, thyroid, and pancreatic cancer [37, 38, 39], and ZDHHC8P1 was able to promote the progression of colon cancer [40]. However, MBL1P, CSNK1A1P, UBE2Q2P1, and BCORP1 were newly proposed m6AlncRNAs in this study, and their potential mechanisms require further investigation. The prognosis of the low‐risk group was significantly better than that of the high‐risk group. The AUC of the 2‐, 3‐, and 5‐year ROC curves were 0.912, 0.844, and 0.824, respectively, showing good predictive value. Further independent prognostic analysis suggested that the model was an independent prognostic factor for 46 patients. In addition, through correlation analysis, we also found that the high‐and low‐risk groups were highly consistent with the cluster grouping in clinical characteristics and immune characteristics. The low‐risk group was part of Cluster 1 with an earlier tumor stage and N stage, and a lower risk score indicated a lower risk of metastasis (M1). The nomogram based on the model and tumor staging showed better predictive value, with the AUC of the 2‐, 3‐, and 5‐year ROC curves being 0.924, 0.930, and 0.866, respectively, and the C‐index was 0.829.

To explore the clinical application value of the model, we performed an analysis of the immune characteristics and chemosensitivity. The expression of PD‐L1 was higher in Cluster 2 and high‐risk groups, with no difference in the expression of PD‐1. Memory B cell, Tregs, and CD4 memory T cell activation display a characteristic infiltration. Cancer cells' escape from T cell‐mediated immune surveillance accelerates the proliferation of tumor cells, enhances the infiltration ability of tumor cells [41], and acts as an important factor affecting the outcome of cancer immunotherapy [42, 43, 44]. Since Cluster 1 and low‐risk groups show high infiltration of Tregs, drugs targeting specific targets of Tregs, such as CCR4 [45] and CCR8 [46, 47, 48], may benefit these groups. On the other hand, immunotherapy targeting PD‐L1 may benefit Cluster 2 with high‐risk PD‐L1 expression [49]. Using the Cancer Genome Project drug prediction database, drug susceptibility analysis of ‘driver‐gene‐negative’ LUAD was performed to seek available treatment. We found that the low‐risk group was more suitable for treatment with first‐line chemotherapeutic agents, such as docetaxel, gemcitabine, or paclitaxel. These results further proved that the expression of m6AlncRNAs can indicate the immune characteristics of patients with ‘driver‐gene‐negative’ LUAD, and the classification of patients in this study may assist clinicians in the selection of immunotherapeutic and chemotherapeutic agents.

Based on the nine m6AlncRNAs, a pan‐cancer analysis of 33 cancers in TCGA was performed to discover the tumor heterogeneity of key m6AlncRNAs in different cancers. SDHAP1, ZDHHC8P1, and DIO3OS were significantly differentially expressed in several cancer types. Interestingly, DIO3OS was validated by FISH experiments in tissues from patients with ‘driver‐gene‐negative’ LUAD and also found to be closely associated with ESTIMATE, stromal/immune, and nucleic acid stemness scores. DIO3OS may be the most important key m6AlncRNA in the development of many cancers. Many studies have shown that low DIO3OS expression promotes the occurrence and progression of cancers such as hepatocellular carcinoma, pancreatic carcinoma, and esophageal squamous cell carcinoma [37, 39, 50, 51]. A recent study also suggested that CpG456 methylation downregulated DIO3OS expression leading to tumorigenesis and metastasis in NSCLC, which indicates that it might exhibit the similar CpG456 methylation in ‘driver‐gene‐negative’ LUAD [52].

This study still has some shortcomings. Because the patients with ‘driver‐gene‐negative’ used in this study lack similar samples in other centers and public databases, there were no externally validated assays performed for this study, which may cause the AUC values reported in Fig. 2D are overly optimistic. There is also a risk of missing variables that were preselected based on univariate models. At the same time, it is difficult to obtain the samples in this study, resulting in that only small sample data from a single center are included in the study, which was cause partial overfitting of the model.

The subtype with ‘driver‐gene‐negative’ is a weak link in the treatment of patients with LUAD. We have preliminarily found an internal association of m6A at the molecular level in these patients, which provides a basis for treatment and prognosis assessment. Further multicenter and large‐sample in‐depth studies are necessary, which will be carried out in our follow‐up work.

## 5 Conclusions

m6AlncRNAs may be involved in the development of ‘driver‐gene‐negative’ LUAD, affecting immune composition and leading to a different prognosis. A signature constructed using nine m6AlncRNAs can effectively predict the prognosis of patients with ‘driver‐gene‐negative’ LUAD. This is an effective complement to TNM stage‐based prognostic judgment and will contribute to the clinical treatment of patients with ‘driver‐gene‐negative’ LUAD.

## Conflict of interest

The authors declare no conflict of interest.

## Author contributions

HY, HC, SS, TC, JZ and MA conceived and designed the project, HY, HC and YZ wrote the article, and HL, YL, ZK, and YZ revised the article.

### Peer review

The peer review history for this article is available at https://publons.com/publon/10.1002/1878-0261.13323.

## Supporting information


**Fig. S1.** The detection of PD‐1 and PD‐L1 in “driver‐gene‐negative” LUAD by immunohistochemical staining. 6 patients were randomly selected from the high‐risk and low‐risk groups for immunohistochemical analysis. The expression of PD‐1 and PD‐L1 was divided into four grades by the depth of staining. The differences in the expression of immune checkpoint genes between the two groups were compared.

**Table S1.** Clinical information of included 46 patients with “driver‐genenegative” LUAD.

## Data Availability

The data that support the findings of this study are available from the corresponding author zhuy45@mail.sysu.edu.cn upon reasonable request.
