# Supplementary figures and images for: Methylation of N6 adenosine‐related long noncoding RNA: effects on prognosis and treatment in ‘driver‐gene‐negative’ lung adenocarcinoma
Source: Mol Oncol. 2022 Nov 16;17(2):365–77. doi: 10.1002/1878-0261.13323 (PMC9892826; doi:10.1002/1878-0261.13323)

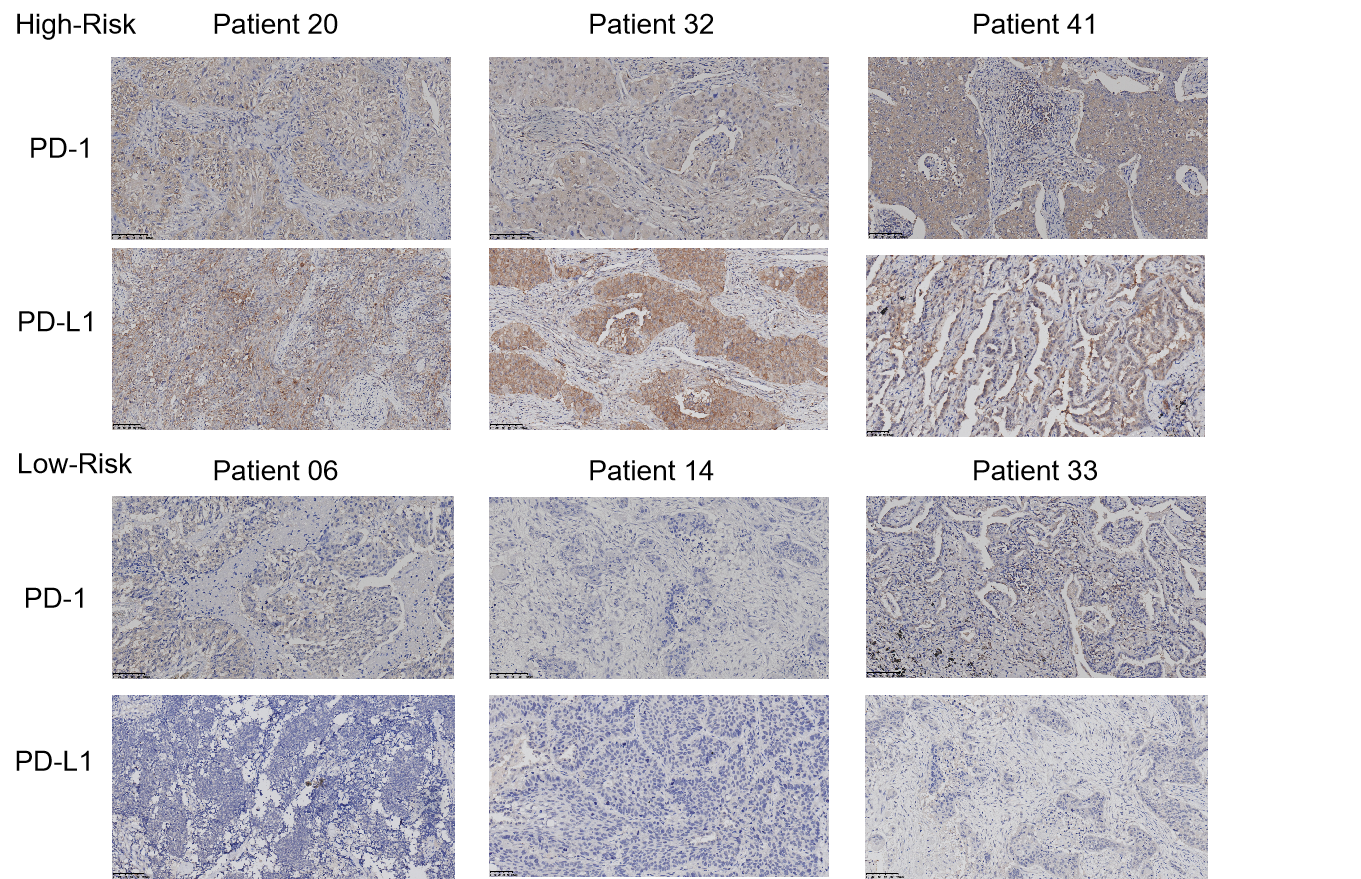

Supplement: Supplementary file 1 — Fig. S1. The detection of PD‐1 and PD‐L1 in “driver‐gene‐negative” LUAD by immunohistochemical staining. 6 patients were randomly selected from the high‐risk and low‐risk groups for immunohistochemical analysis. The expression of PD‐1 and PD‐L1 was divided into four grades by the depth of staining. The differences in the expression of immune checkpoint genes between the two groups were compared. [file MOL2-17-365-s002.bmp]
