# Supplementary material for: Methylation of N6 adenosine‐related long noncoding RNA: effects on prognosis and treatment in ‘driver‐gene‐negative’ lung adenocarcinoma
Source: Mol Oncol. 2022 Nov 16;17(2):365–77. doi: 10.1002/1878-0261.13323 (PMC9892826; doi:10.1002/1878-0261.13323)
Supplement: Supplementary file 2 — Table S1. Clinical information of included 46 patients with “driver‐genenegative” LUAD. [file MOL2-17-365-s001.docx]

**Supplement Table** Clinical information of included 46 patients with “driver-gene-negative” LUAD

| No. | Gender | Age | Stage | TNM | OS(months) | Status |
| --- | --- | --- | --- | --- | --- | --- |
| 1 | Male | 72 | Ⅰ | T1N0M0 | 66 | Alive |
| 2 | Male | 53 | Ⅰ | T2N0M0 | 49 | Alive |
| 3 | Male | 45 | Ⅰ | T1N0M0 | 42 | Dead |
| 4 | Male | 42 | Ⅰ | T1N0M0 | 102 | Alive |
| 5 | Female | 73 | Ⅰ | T1N0M0 | 48 | Alive |
| 6 | Female | 66 | Ⅰ | T1N0M0 | 61 | Dead |
| 7 | Female | 47 | Ⅰ | T1N0M0 | 58 | Dead |
| 8 | Female | 45 | Ⅰ | T2N0M0 | 60 | Dead |
| 9 | Male | 68 | Ⅰ | T2N0M0 | 79 | Dead |
| 10 | Male | 74 | Ⅰ | T1N0M0 | 49 | Alive |
| 11 | Male | 65 | Ⅰ | T1N0M0 | 39 | Alive |
| 12 | Male | 52 | Ⅰ | T1N0M0 | 21 | Alive |
| 13 | Male | 65 | Ⅰ | T2N0M0 | 50 | Dead |
| 14 | Male | 62 | Ⅱ | T2N1M0 | 46 | Alive |
| 15 | Female | 35 | Ⅱ | T3N0M0 | 49 | Alive |
| 16 | Female | 53 | Ⅱ | T1N0M0 | 49 | Dead |
| 17 | Female | 49 | Ⅱ | T2N1M0 | 39 | Alive |
| 18 | Male | 70 | Ⅱ | T3N0M0 | 26 | Alive |
| 19 | Male | 59 | Ⅱ | T3N0M0 | 29 | Alive |
| 20 | Male | 52 | Ⅱ | T2N1M0 | 17 | Alive |
| 21 | Female | 56 | Ⅱ | T1N1M0 | 42 | Alive |
| 22 | Female | 38 | Ⅱ | T1N1M0 | 40 | Dead |
| 23 | Male | 65 | Ⅱ | T1N1M0 | 36 | Alive |
| 24 | Female | 59 | Ⅱ | T2N1M0 | 12 | Alive |
| 25 | Female | 66 | Ⅱ | T1N1M0 | 73 | Dead |
| 26 | Female | 53 | Ⅱ | T2N0M0 | 54 | Dead |
| 27 | Female | 57 | Ⅲ | T1N2M0 | 28 | Alive |
| 28 | Male | 59 | Ⅲ | T1N2M0 | 31 | Alive |
| 29 | Male | 76 | Ⅲ | T1N2M0 | 29 | Alive |
| 30 | Female | 56 | Ⅲ | T1N2M0 | 19 | Alive |
| 31 | Female | 61 | Ⅲ | T2N2M0 | 26 | Alive |
| 32 | Male | 51 | Ⅲ | T2N2M0 | 54 | Dead |
| 33 | Male | 46 | Ⅲ | T2N0M0 | 30 | Dead |
| 34 | Female | 63 | Ⅲ | T2N2M0 | 22 | Alive |
| 35 | Female | 59 | Ⅲ | T2N2M0 | 23 | Alive |
| 36 | Female | 38 | Ⅲ | T2N2M0 | 24 | Alive |
| 37 | Female | 58 | Ⅲ | T1N3M0 | 39 | Alive |
| 38 | Female | 61 | Ⅳ | T4N3M1 | 20 | Alive |
| 39 | Female | 48 | Ⅳ | T2N3M1 | 32 | Alive |
| 40 | Male | 48 | Ⅳ | T4N1M0 | 21 | Alive |
| 41 | Male | 62 | Ⅳ | T3N2M1 | 19 | Alive |
| 42 | Female | 28 | Ⅳ | T1N3M1 | 8 | Alive |
| 43 | Male | 68 | Ⅳ | T4N3M1 | 18 | Alive |
| 44 | Female | 68 | Ⅳ | T2N2M1 | 16 | Alive |
| 45 | Female | 57 | Ⅳ | T4N3M1 | 13 | Alive |
| 46 | Male | 46 | Ⅳ | T1N2M1 | 18 | Dead |
